# Supplementary figures and images for: Characterization of the transcriptional response of Candida parapsilosis to the antifungal peptide MAF-1A
Source: PeerJ. 2020 Sep 7;8:e9767. doi: 10.7717/peerj.9767 (PMC7482638; doi:10.7717/peerj.9767)

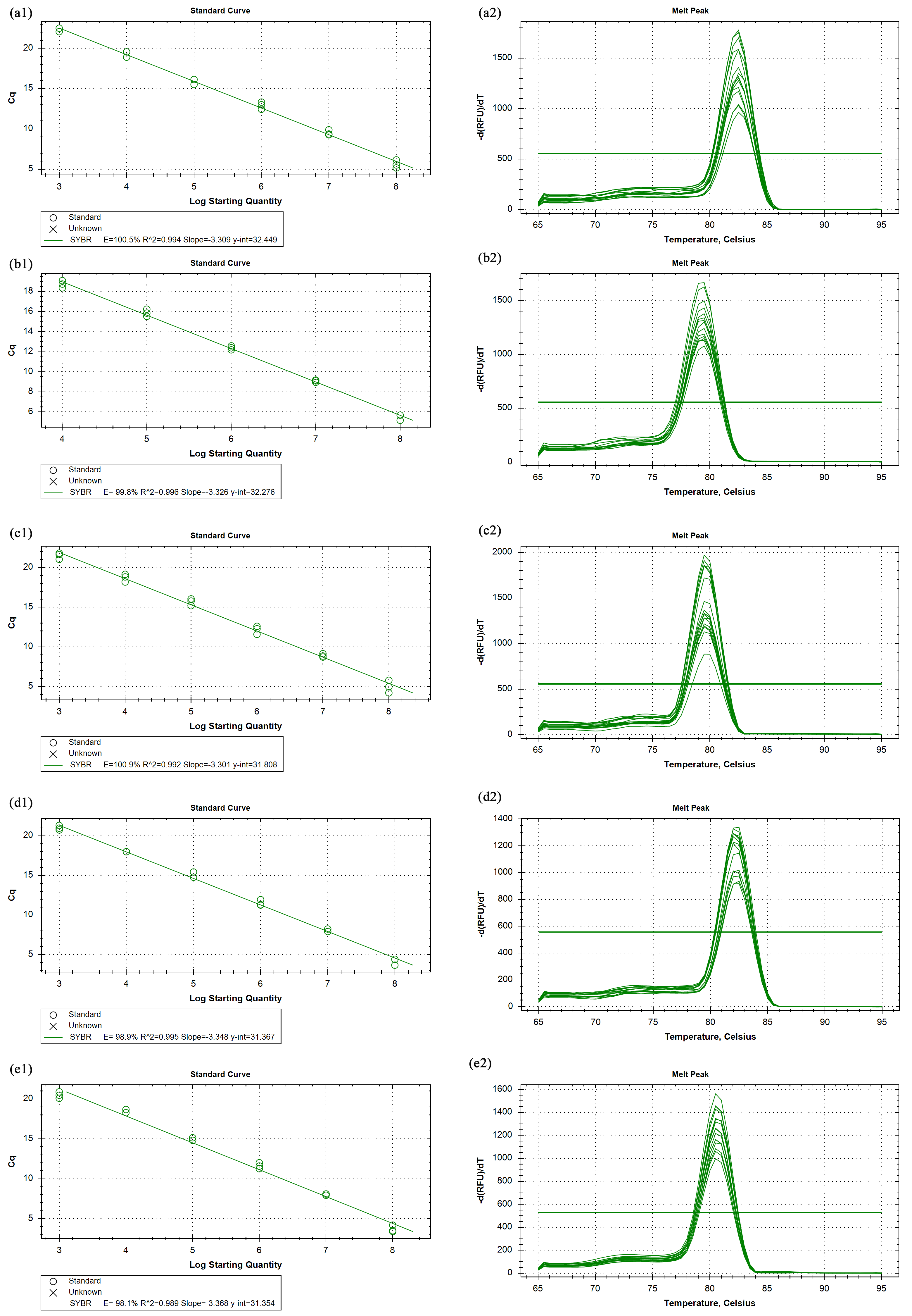

Supplement: Figure S1 — CPAR2_208190 (a1, a2), CPAR2_213060 (b1, b2), CPAR2_203780 (c1, c2), CPAR2_404910 (d1, d2), CPAR2_800950 (e1, e2). [file peerj-08-9767-s001.png]

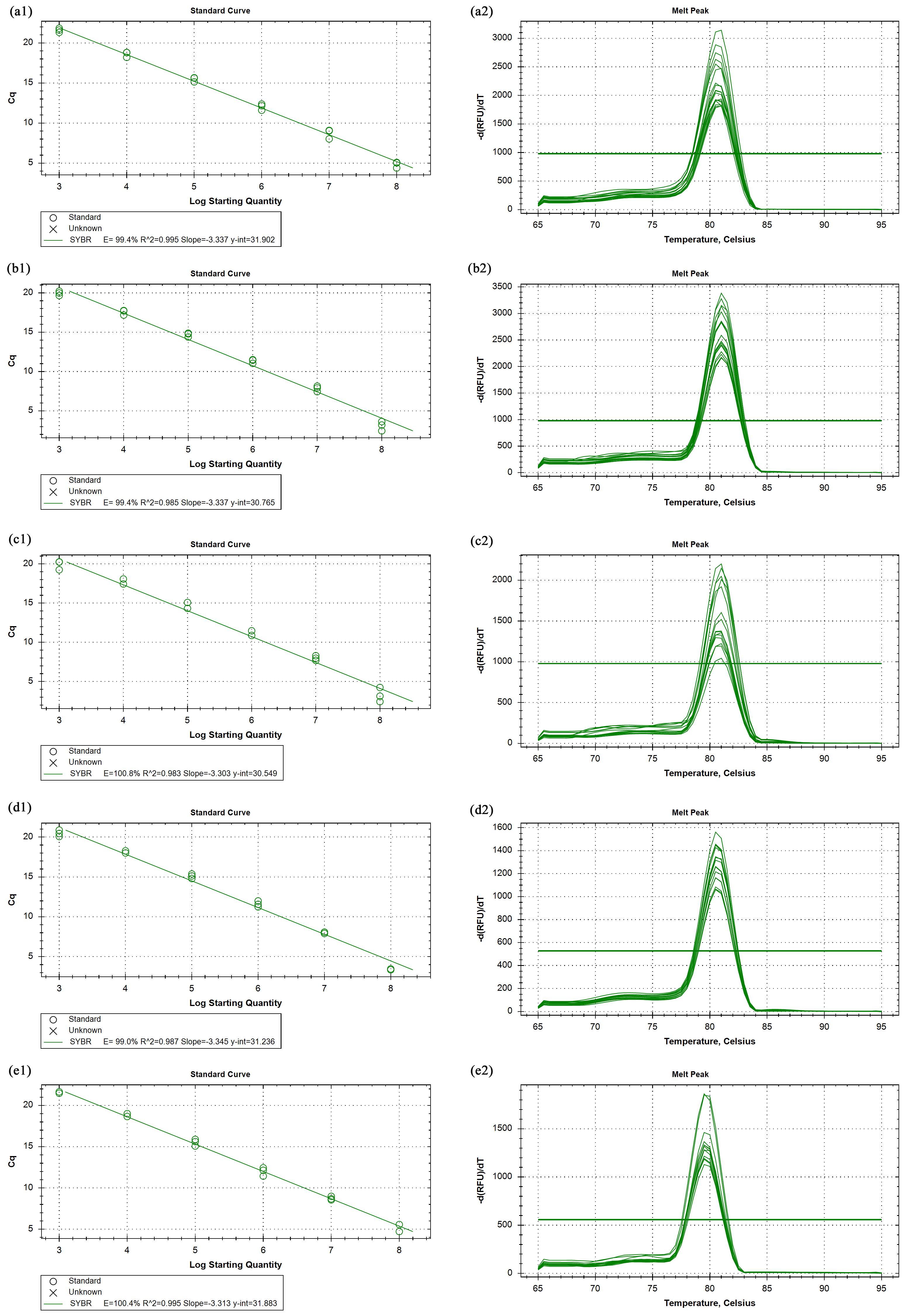

Supplement: Figure S2 — CPAR2_702930 (a1, a2), CPAR2_807710 (b1, b2), CPAR2_703200 (c1, c2), CPAR2_807700 (d1, d2), CPAR2_700300 (e1, e2). [file peerj-08-9767-s002.png]

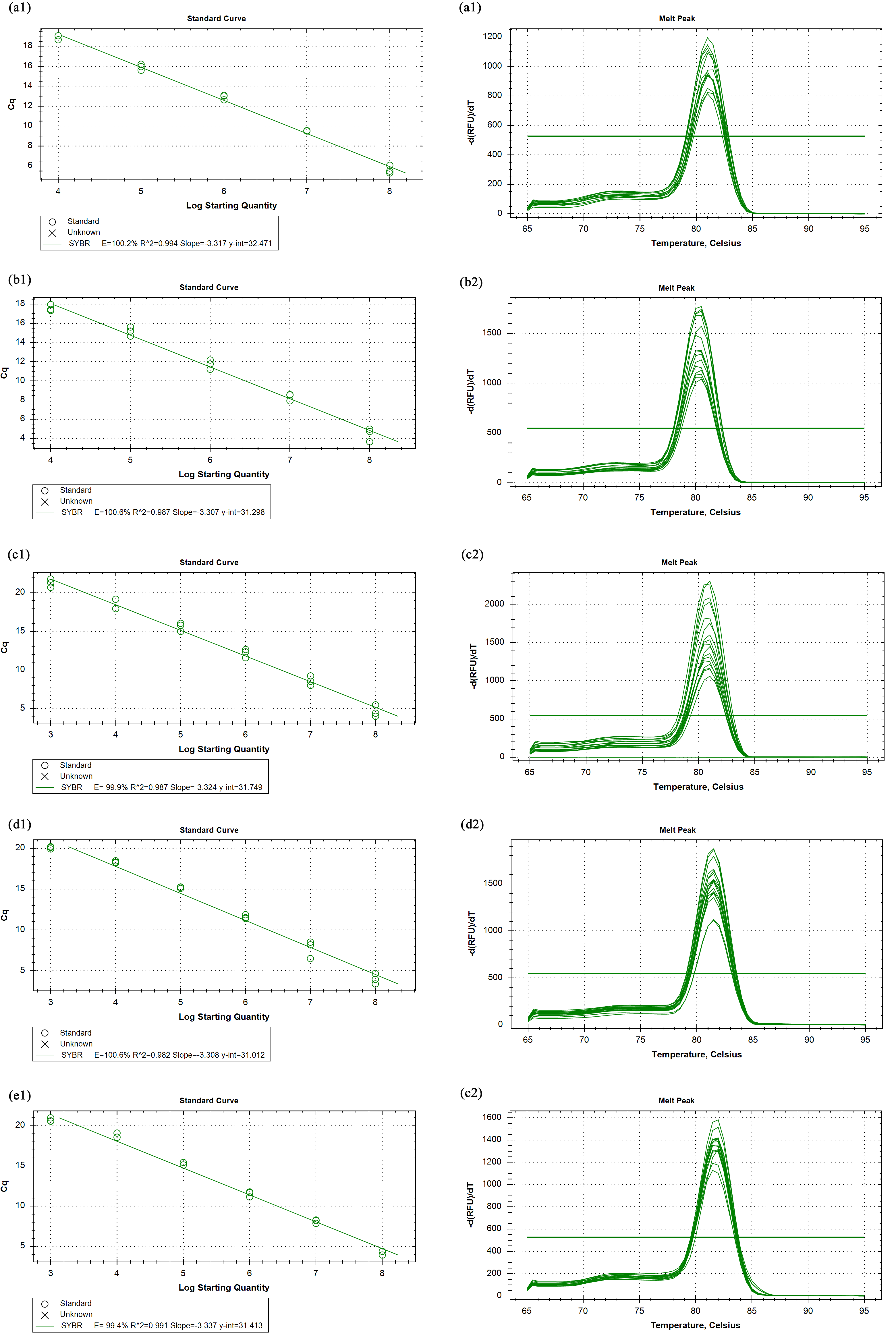

Supplement: Figure S3 — CPAR2_100480(a1, a2), CPAR2_603600 (b1, b2), CPAR2_808120 (c1, c2), CPAR2_102580 (d1, d2), CPAR2_109900 (e1, e2). [file peerj-08-9767-s003.png]

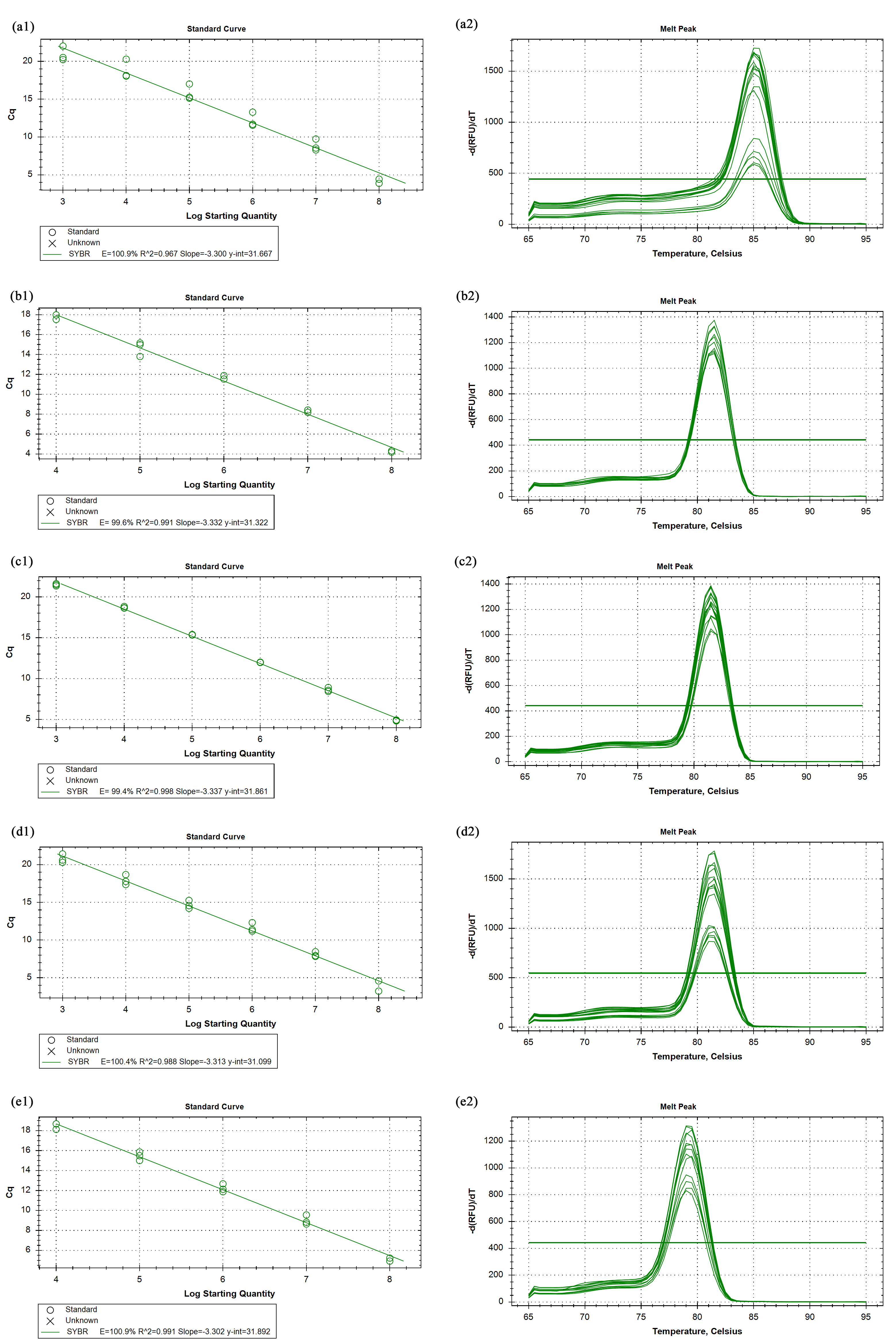

Supplement: Figure S4 — CPAR2_603040 (a1, a2), CPAR2_403560 (b1, b2), CPAR2_202420 (c1, c2), CPAR2_602060 (d1, d2), CPAR2_109200 (e1, e2). [file peerj-08-9767-s004.png]

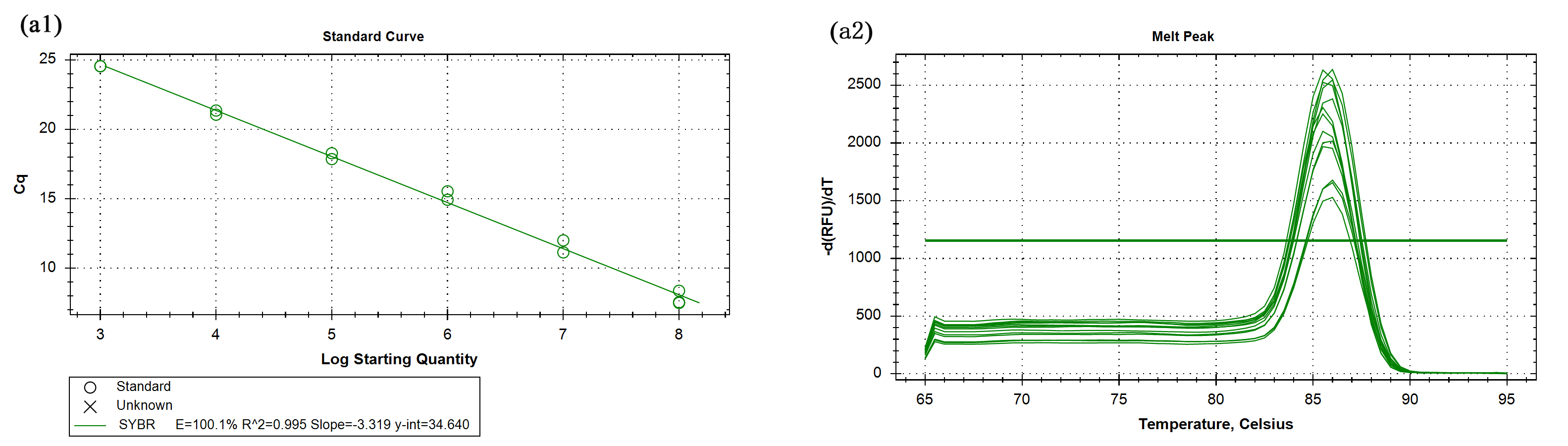

Supplement: Figure S5 — 18S rRNA (a1, a2). [file peerj-08-9767-s005.png]

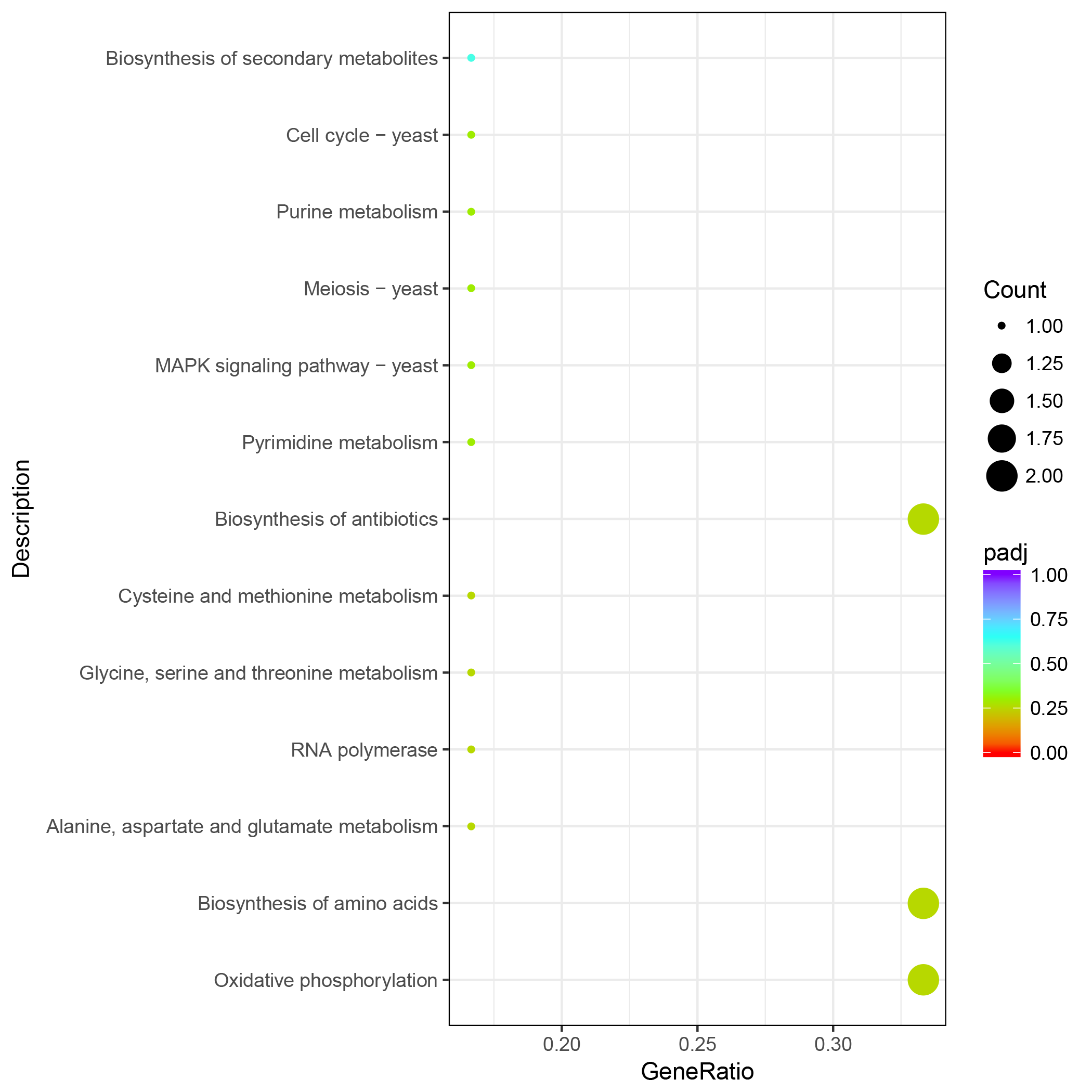

Supplement: Figure S6 — X axis is the ratio of differential genes, and Y axis is the KEGG pathway. [file peerj-08-9767-s006.png]
