# Supplementary material for: Characterization of the transcriptional response of Candida parapsilosis to the antifungal peptide MAF-1A
Source: PeerJ. 2020 Sep 7;8:e9767. doi: 10.7717/peerj.9767 (PMC7482638; doi:10.7717/peerj.9767)
Supplement: Table S1 — F: forward; R: reverse. [file peerj-08-9767-s007.docx]

Table S1.Primer sequences. F: forward; R: reverse.

| Gene | Primers sequences | PCR product length (bp) |
| --- | --- | --- |
| CPAR2_208190 | F:TTGGGTTTGGGTGTGTTCCA | 196 |
|  | R:TGGCCGAAATGTCTGACGAA |  |
| CPAR2_213060 | F:GTCGCAAATTGGCTTAGCTACT | 102 |
|  | R:GGTTTCATTGTTGGGGATTCGG |  |
| CPAR2_203780 | F:GCGTCCTCAATTTGCAAGGG | 223 |
|  | R:TGCAACTTCGGGGTCACATT |  |
| CPAR2_404910 | F:TGCCAAGGGTCCAGAGTACT | 217 |
|  | R:AACGGCAAGGAAGGAAACAC |  |
| CPAR2_800950 | F:AGCACCAATAGTACCAGCAGT | 150 |
|  | R:TTAGCTGCATTCACCACTACT |  |
| CPAR2_702930 | F:TGGCACAGGTACAGATGGTT | 186 |
|  | R:TTGAGGTTGTTCAGGTGGCA |  |
| CPAR2_807710 | F:GTGGTCACGGTTACTCAGCA | 165 |
|  | R:CTCCCTTAACAGCCTTGCCA |  |
| CPAR2_703200 | F:AGCTTTTGTCTTGGCTCCCA | 211 |
|  | R:AGTGACTATGGCGTTTGGGT |  |
| CPAR2_807700 | F:TGCTGCCACTGTTGTTTTGG | 249 |
|  | R:CGTGGAGGGTTCAAGGTCTT |  |
| CPAR2_700300 | F:TGTTGTCAAGATCCCAGGTGG | 202 |
|  | R:TGGGGTTTCATCTTCAGGGG |  |
| CPAR2_100480 | F:TCTTATGCTGCGGTTGCTGG | 97 |
|  | R:CCAAAGCCTGCTCGTATGTGA |  |
| CPAR2_603600 | F:CCGCTGGTGATGAGGCTACTAATG | 136 |
|  | R:TGACCGTGCAAGTGGAATGGATG |  |
| CPAR2_808120 | F:TGGCAATCACATCCGTTCACCTTC | 163 |
|  | R:CGTATGGACCTTCGCAACTCACTC |  |
| CPAR2_102580 | F:CCAGCACCTCGGTCAATCAAGG | 159 |
|  | R:CAGTAGCTGCACCTGGTGAGTTC |  |
| CPAR2_109900 | F:AGCAGCACATTCCGCACCAAG | 117 |
|  | R:GCGACAGGAGCATGGTCATGG |  |
| CPAR2_603040 | F:CACGTCGTGGAGGACACTCAAG | 81 |
|  | R:CTGAATGGCTGCCGCTGGAG |  |
| CPAR2_403560 | F:CAAGATAGCTGTGCAACGTTCACC | 131 |
|  | R:ATGGACGCACCGACAAGTTGG |  |
| CPAR2_202420 | F:CAGGACCTGAAGGAACGTCTCAAC | 155 |
|  | R:TTCGGATGCTGTCTCGCTTGATG |  |
| CPAR2_602060 | F:CCGTCAGCATCACCATGCCAAG | 137 |
|  | R:GTGAGGCTGTTGTGAAGCTAGGTG |  |
| CPAR2_109200 | F:GCCGAGATCACCTCCATTGCATAG | 89 |
|  | R:TAGCATTAGCTGCGTGTGACGAAG |  |
