# Supplementary material for: Characterization of the transcriptional response of Candida parapsilosis to the antifungal peptide MAF-1A
Source: PeerJ. 2020 Sep 7;8:e9767. doi: 10.7717/peerj.9767 (PMC7482638; doi:10.7717/peerj.9767)
Supplement: Table S2 — reference gene: 18S rRNA. [file peerj-08-9767-s008.docx]

Table S2.The primer efficiency of 20 differentially expressed genes and reference gene (18S rRNA).

| Gene_name | primer efficiency |
| --- | --- |
| CPAR2_208190 | 100.5% |
| CPAR2_213060 | 99.8% |
| CPAR2_203780 | 100.9% |
| CPAR2_404910 | 98.9% |
| CPAR2_800950 | 98.1% |
| CPAR2_702930 | 99.4% |
| CPAR2_807710 | 99.4% |
| CPAR2_703200 | 100.8% |
| CPAR2_807700 | 99.0% |
| CPAR2_700300 | 100.4% |
| CPAR2_100480 | 100.2% |
| CPAR2_603600 | 100.6% |
| CPAR2_808120 | 99.9% |
| CPAR2_102580 | 100.6% |
| CPAR2_109900 | 99.4% |
| CPAR2_603040 | 100.9% |
| CPAR2_403560 | 99.6% |
| CPAR2_202420 | 99.4% |
| CPAR2_602060 | 100.4% |
| CPAR2_109200 | 100.9% |
| 18S rRNA | 100.1% |
