# Supplementary material for: Characterization of the transcriptional response of Candida parapsilosis to the antifungal peptide MAF-1A
Source: PeerJ. 2020 Sep 7;8:e9767. doi: 10.7717/peerj.9767 (PMC7482638; doi:10.7717/peerj.9767)
Supplement: Table S3 — the C. parapsilosis were treated with MAF-1A at MIC for 6 h (CPAS) and 18h (CPBS). The untreated cultures CPAC and CPBC as control; CA_DT: the C. albicans were treated with MAF-1A at MIC for 2 h, CA_D: untreated cultures. [file peerj-08-9767-s009.docx]

Table S3. A comparison between the DEGs of C. albicans and C. parapsilosis to MAF-1A. the C. parapsilosis were treated with MAF-1A at MIC for 6 h (CPAS) and 18h (CPBS). The untreated cultures CPAC and CPBC as control; CA_DT: the C. albicans were treated with MAF-1A at MIC for 2 h, CA_D: untreated cultures.

|  | CPAS vs CPAC | | CPBS vs CPBC | |  | CA_DT vs CA_D | |
| --- | --- | --- | --- | --- | --- | --- | --- |
| Gene Name | Log2 FC | P value | Log2 FC | P value | Homologs | Log2 FC | P value |
| CPAR2_208190 | -2.05 | 1.68×10-^18^ | 0.12 | 3.95×10^-1^ | PHO84 | -2.10 | 9.42×10-^7^ |
| CPAR2_213060 | -1.82 | 2.13×10-^24^ | -0.05 | 7.50×10-^1^ | no | / | / |
| CPAR2_203780 | -1.54 | 5.43×10-^60^ | 0.06 | 6.14×10-^1^ | CAALFM_C603600CA | 9.22 | 1.52×10-^6^ |
| CPAR2_404910 | -1.51 | 2.27×10-^4^ | 0.62 | 2.78×10-^2^ | CAALFM_C302070CA | 0.16 | 7.81×10-1 |
| CPAR2_800950 | -1.48 | 1.02×10-^41^ | 0.70 | 1.63×10-^11^ | CAALFM_CR00910WA | -1.41 | 7.53×10^-4^ |
| CPAR2_700300 | 1.76 | 2.81×10-^114^ | 0.26 | 4.58×10-^1^ | CAALFM_C114020WA | 0.55 | 2.24×10-^1^ |
| CPAR2_807700 | 1.50 | 2.23×10^-71^ | 0.14 | 7.32×10-^1^ | POX1-3 | 2.51 | 7.01×10^-9^ |
| CPAR2_703200 | 1.47 | 6.62×10-^14^ | -0.19 | 1.04×10-^1^ | LIP4 | 0.96 | 2.45×10^-2^ |
| CPAR2_807710 | 1.29 | 2.17×10-^86^ | 0.12 | 6.38×10-^1^ | POX1-3 | 2.51 | 7.01×10^-9^ |
| CPAR2_702930 | 1.28 | 9.38×10-^7^ | 0.15 | 4.12×10-^1^ | CAALFM_C702360WA | -0.56 | 8.15×10^-1^ |
| CPAR2_100480 | -0.02 | 9.39×10-^1^ | -1.25 | 1.22×10-^6^ | GRP2 | -2.82 | 1.45×10^-10^ |
| CPAR2_603600 | -0.48 | 1.78×10-^10^ | -0.86 | 1.56×10-^15^ | FET31 | 0.11 | 7.86×10^-1^ |
| CPAR2_808120 | -1.12 | 2.11×10^-43^ | -0.84 | 5.97×10-^27^ | CFL5 | 2.99 | 2.42×10-^5^ |
| CPAR2_102580 | -0.47 | 4.43×10^-12^ | -0.70 | 4.98×10-^17^ | SAP8 | / | / |
| CPAR2_109900 | 0.70 | 2.78×10^-12^ | -0.54 | 1.84×10-^8^ | NRM1 | -0.36 | 4.23×10-^1^ |
| CPAR2_603040 | 0.54 | 3.09×10-15 | 0.64 | 2.00×10-16 | no | / | / |
| CPAR2_403560 | 0.26 | 3.03×10-3 | 0.58 | 1.65×10-11 | CAALFM_C403580WA | 1.11 | 8.38×10-^3^ |
| CPAR2_202420 | 1.15 | 1.26×10-89 | 0.57 | 9.60×10-7 | TPO4 | 1.05 | 1.32×10-^2^ |
| CPAR2_602060 | 0.16 | 2.62×10-2 | 0.52 | 6.69×10-13 | ZCF18 | -0.06 | 8.83×10^-1^ |
| CPAR2_109200 | 0.37 | 1.14×10^-7^ | 0.50 | 7.52×10^-11^ | CAALFM_C101400CA | 0.53 | 1.99×10^-1^ |
