# Supplementary material for: Characterization of the transcriptional response of Candida parapsilosis to the antifungal peptide MAF-1A
Source: PeerJ. 2020 Sep 7;8:e9767. doi: 10.7717/peerj.9767 (PMC7482638; doi:10.7717/peerj.9767)
Supplement: Table S4 — The C. parapsilosis were treated with MAF-1A at MIC for 6 h (CPAS) and 18h (CPBS), untreated cultures CPAC and CPBC as control; CA_DT: the C. albicans were treated with MAF-1A at MIC for 2 h, CA_D: untreated cultures. [file peerj-08-9767-s010.docx]

Table S4. A comparison between the significantly enriched KEGG pathways for DEGs of C. albicans and C. parapsilosis to MAF-1A. The C. parapsilosis were treated with MAF-1A at MIC for 6 h (CPAS) and 18h (CPBS), untreated cultures CPAC and CPBC as control; CA_DT: the C. albicans were treated with MAF-1A at MIC for 2 h, CA_D: untreated cultures.

| Group | KEGGID | Description | padj | Data resources |
| --- | --- | --- | --- | --- |
| CPASvsCPAC_up | cdu00190 | Oxidative phosphorylation | 2.84×10-10 | Candida parapsilosis (this study) |
|  | cdu04146 | Peroxisome | 8.56×10-7 |  |
|  | cdu00020 | Citrate cycle (TCA cycle) | 6.27×10-4 |  |
|  | cdu01200 | Carbon metabolism | 7.44×10-4 |  |
|  | cdu04111 | Cell cycle - yeast | 1.56×10-3 |  |
|  | cdu04011 | MAPK signaling pathway - yeast | 1.16×10-2 |  |
|  | cdu04113 | Meiosis - yeast | 2.80×10-2 |  |
|  | cdu00071 | Fatty acid degradation | 2.80×10-2 |  |
|  | cdu04136 | Autophagy - other | 3.80×10-2 |  |
| CPBSvsCPBC_up | cdu00220 | Arginine biosynthesis | 7.21×10-3 | Candida parapsilosis (this study) |
|  | cdu00250 | Alanine, aspartate and glutamate metabolism | 1.77×10-2 |  |
| CA_DTvsCA_D_up | cal04146 | Peroxisome | 2.80×10-12 | Candida albicans (Wang et al. 2017) |
|  | cal00190 | Oxidative phosphorylation | 2.80×10-12 |  |
|  | cal03010 | Ribosome | 2.26×10-11 |  |
|  | cal00071 | Fatty acid degradation | 2.61×10-10 |  |
|  | cal00410 | beta-Alanine metabolism | 3.97×10-6 |  |
|  | cal01200 | Carbon metabolism | 7.38×10-6 |  |
|  | cal00280 | Valine, leucine and isoleucine degradation | 7.77×10-5 |  |
|  | cal00640 | Propanoate metabolism | 8.92×10-5 |  |
|  | cal00630 | Glyoxylate and dicarboxylate metabolism | 5.63×10-4 |  |
|  | cal00020 | Citrate cycle (TCA cycle) | 7.67×10-4 |  |
|  | cal00330 | Arginine and proline metabolism | 1.03×10-3 |  |
|  | cal00250 | Alanine, aspartate and glutamate metabolism | 3.31×10-3 |  |
|  | cal01212 | Fatty acid metabolism | 4.58×10-3 |  |
|  | cal00220 | Arginine biosynthesis | 3.29×10-2 |  |
|  | cal01040 | Biosynthesis of unsaturated fatty acids | 3.29×10-2 |  |
| CPASvsCPAC_down | cdu00100 | Steroid biosynthesis | 1.40×10-6 | Candida parapsilosis (this study) |
|  | cdu01230 | Biosynthesis of amino acids | 2.53×10-5 |  |
|  | cdu00270 | Cysteine and methionine metabolism | 1.36×10-4 |  |
|  | cdu01130 | Biosynthesis of antibiotics | 2.27×10-4 |  |
|  | cdu03010 | Ribosome | 5.12×10-4 |  |
|  | cdu03020 | RNA polymerase | 3.03×10-3 |  |
|  | cdu01110 | Biosynthesis of secondary metabolites | 4.47×10-3 |  |
|  | cdu03013 | RNA transport | 8.83×10-3 |  |
|  | cdu03008 | Ribosome biogenesis in eukaryotes | 8.83×10-3 |  |
|  | cdu00300 | Lysine biosynthesis | 1.96×10-2 |  |
|  | cdu01210 | 2-Oxocarboxylic acid metabolism | 2.05×10-2 |  |
|  | cdu00240 | Pyrimidine metabolism | 2.40×10-2 |  |
|  | cdu00260 | Glycine, serine and threonine metabolism | 2.40×10-2 |  |
|  | cdu00230 | Purine metabolism | 3.09×10-2 |  |
| CPBSvsCPBC_down | cdu01200 | Carbon metabolism | 7.61×10-7 | Candida parapsilosis (this study) |
|  | cdu01130 | Biosynthesis of antibiotics | 1.43×10-5 |  |
|  | cdu00190 | Oxidative phosphorylation | 6.91×10-5 |  |
|  | cdu01110 | Biosynthesis of secondary metabolites | 6.91×10-5 |  |
|  | cdu00010 | Glycolysis / Gluconeogenesis | 1.57×10-4 |  |
|  | cdu00260 | Glycine, serine and threonine metabolism | 4.47×10-3 |  |
|  | cdu01230 | Biosynthesis of amino acids | 1.16×10-2 |  |
|  | cdu00680 | Methane metabolism | 2.05×10-2 |  |
|  | cdu00520 | Amino sugar and nucleotide sugar metabolism | 2.05×10-2 |  |
|  | cdu00052 | Galactose metabolism | 2.87×10-2 |  |
|  | cdu00730 | Thiamine metabolism | 3.20×10-2 |  |
|  | cdu00630 | Glyoxylate and dicarboxylate metabolism | 3.20×10-2 |  |
|  | cdu00330 | Arginine and proline metabolism | 3.24×10-2 |  |
|  | cdu00670 | One carbon pool by folate | 3.24×10-2 |  |
| CA_DTvsCA_D_down | cal03050 | Proteasome | 1.45×10-6 | Candida albicans (Wang et al. 2017) |
|  | cal01110 | Biosynthesis of secondary metabolites | 1.16×10-5 |  |
|  | cal00010 | Glycolysis / Gluconeogenesis | 9.67×10-3 |  |
|  | cal00230 | Purine metabolism | 1.19×10-2 |  |
|  | cal00920 | Sulfur metabolism | 2.04×10-2 |  |
|  | cal00620 | Pyruvate metabolism | 2.04×10-2 |  |
|  | cal01200 | Carbon metabolism | 2.04×10-2 |  |
