# Supplementary material for: Characterization of the transcriptional response of Candida parapsilosis to the antifungal peptide MAF-1A
Source: PeerJ. 2020 Sep 7;8:e9767. doi: 10.7717/peerj.9767 (PMC7482638; doi:10.7717/peerj.9767)
Supplement: Data S1 [file peerj-08-9767-s011.zip › raw data/MIC assay (raw data).docx]

**Table R1.** MIC values were determined by MIC assay (raw data). MIC was defined as the lowest concentration resulting in fungus growth inhibition ≥80% compared to the control.

|  | Growth control | MAF-1A | | | | | | | | | |
| --- | --- | --- | --- | --- | --- | --- | --- | --- | --- | --- | --- |
|  |  | 0.1mg/ml | 0.2mg/ml | 0.3mg/ml | 0.4mg/ml | 0.5mg/ml | 0.6mg/ml | 0.7mg/ml | 0.8mg/ml | 0.9mg/ml | 1.0mg/ml |
| OD490nm | 0.297 | 0.251 | 0.19 | 0.178 | 0.139 | 0.123 | 0.061 | 0.044 | 0.025 | 0.021 | 0.019 |
|  | 0.314 | 0.239 | 0.197 | 0.188 | 0.129 | 0.109 | 0.059 | 0.05 | 0.022 | 0.025 | 0.017 |
|  | 0.299 | 0.245 | 0.183 | 0.177 | 0.144 | 0.095 | 0.057 | 0.043 | 0.023 | 0.027 | 0.02 |
| Mean ± standard | 0.303±0.008 | 0.245±0.005 | 0.190±0.006 | 0.181±0.005 | 0.137±0.006 | 0.109±0.011 | 0.059±0.002 | 0.046±0.003 | 0.023±0.001 | 0.024±0.002 | 0.019±0.001 |
| fungus growth percentage | / | 80.77% | 62.64% | 59.67% | 45.27% | 35.93% | 19.45% | 15.05% | 7.69% | 8.02% | 6.15% |
| fungus growth inhibition percentage | / | 19.23% | 37.36% | 40.33% | 54.73% | 64.07% | 80.55% | 84.95% | 92.31% | 91.98% | 93.85% |
